# New insights into trophic aerenchyma formation strategy in maize (*Zea mays* L.) organs during sulfate deprivation

**DOI:** 10.3389/fpls.2014.00581

**Published:** 2014-11-03

**Authors:** Filippa Maniou, Styliani N. Chorianopoulou, Dimitris L. Bouranis

**Affiliations:** Plant Physiology Laboratory, Crop Science Department, Agricultural University of AthensAthens, Greece

**Keywords:** aerenchyma, maize, sulfate deprivation, leaf, mesocotyl, deficiency

## Abstract

Aerenchyma attributes plant tissues that contain enlarged spaces exceeding those commonly found as intracellular spaces. It is known that sulfur (S) deficiency leads to formation of aerenchyma in maize adventitious roots by lysis of cortical cells. Seven-day-old maize plants were grown in a hydroponics setup for 19 days under S deprivation against full nutrition. At day 17 and 26 from sowing (d10 and d19 of the deprivation, respectively), a detailed analysis of the total sulfur and sulfate allocation among organs as well as a morphometric characterization were performed. Apart from roots, in S-deprived plants aerenchyma formation was additionally found in the second leaf and in the mesocotyl, too. The lamina (LA) of this leaf showed enlarged gas spaces between the intermediate and small vascular bundles by lysis of mesophyll cells and to a greater extent on the d10 compared to d19. Aerenchymatous spaces were mainly distributed along the middle region of leaf axis. At d10, –S leaves invested less dry mass with more surface area, whilst lesser dry mass was invested per unit surface area in –S LAs. In the mesocotyl, aerenchyma was located near the scutelar node, where mesocotyl roots were developing. In –S roots, more dry mass was invested per unit length. Our data suggest that trying to utilize the available scarce sulfur in an optimal way, the S-deprived plant fine tunes the existing roots with the same length or leaves with more surface area per unit of dry mass. Aerenchyma was not found in the scutelar node and the bases of the attached roots. The sheaths, the LAs’ bases and the crown did not form aerenchyma. This trophic aerenchyma is a localized one, presumably to support new developing tissues nearby, by induced cell death and recycling of the released material. Reduced sulfur allocation among organs followed that of dry mass in a proportional fashion.

## INTRODUCTION

In maize, programmed cell death (PCD) occurs both as a normal process during development as well as in response to environmental stresses and the locations of cell-death events in this species have been reviewed (Buckner et al., 1998, 2000). Especially in response to oxygen deficiency, the cortical cells of the root and stem base can undergo cell death to produce lysigenous aerenchyma. Aerenchyma is tissue containing intercellular spaces that aids the transfer of oxygen from the stem to the root.

Konings and Verschuren (1980) first reported that growth of maize in aerated, N-deficient nutrient solution resulted in the development of aerenchyma in root cortex. Later studies in maize confirmed that both low concentrations of N, P, K, or S nutrition induced aerenchyma formation in root cortex (Drew et al., 1989; Bouranis et al., 2003; Fan et al., 2003; Visser and Voesenek, 2004; Postma and Lynch, 2011a). Therefore, nutrient deficiency stimulates aerenchyma formation in crown roots (CR) of maize. It has been shown that under these deficiencies, root cortical aerenchyma (RCA) does not form in the root base (Siyiannis et al., 2012), a fact which suggests that this aerenchyma is not produced in order to transfer oxygen from the stem to the root. RCA converts living cortical tissue to void volume via PCD. This trophic aerenchyma lowers the respiration of root segments and mobilizes nutrients for other uses (Fan et al., 2003; Postma and Lynch, 2011a; Siyiannis et al., 2012; York et al., 2013). RCA is a root phene, i.e., a unit of root phenotype that affects resource acquisition or utilization (York et al., 2013). Accorcing to Hu et al. (2014), RCA induced by nutrient deficiency in species adapted to aerobic soil conditions, is an adaptive response that reduces root maintenance requirements; in this way greater soil exploration is permitted. Data of Hu et al. (2014) support the hypothesis that RCA can reduce radial transport of some nutrients in some genotypes, an important tradeoff of this trait. A functional–structural model (SimRoot) has been used to provide quantitative support for the hypothesis that RCA formation is a useful adaptation to suboptimal availability of phosphorus, nitrogen, and potassium by reducing the metabolic costs of soil exploration in maize. According to Postma and Lynch (2011a,b), the functional utility of RCA on low-potassium soils is associated with the fact that root growth in potassium-deficient plants was more carbon limited than in phosphorus- and nitrogen-deficient plants. Compared to potassium-deficient plants, phosphorus-, and nitrogen-deficient plants allocate more carbon to the root system as the deficiency develops. On the other hand, on low-phosphorus soils, the utility of RCA was greater in plants with increased lateral branching density than in plants with normal branching. These authors suggest that the large genetic variation in RCA formation, as well as the utility of RCA for a range of stresses render this trait as an interesting crop-breeding target for enhanced soil resource acquisition.

The exact mechanisms that trigger the formation of RCA in maize under nitrate, phosphate, or sulfate deprivation are still unclear (for a review see Bouranis et al., 2007b). Siyiannis et al. (2012) have compared aerenchyma distribution across the first whorl of CR, which were subject to S, N, or P deprivation over a period of 10 days in connection with oxygen consumption and ATP concentration in the whole root. Aerenchyma was not found in the root base regardless of the deprivation. PCD was observed near the root tip, either within the first 2 days (–N) or a few days later (–S, –P) of the treatment. Roots at day 6 under all three nutrient-deprived conditions showed signs of PCD 1 cm behind the cap, whereas only N-deprived root cells 0.5 cm behind the cap showed severe ultrastructural alterations, due to advanced PCD. It has been suggested that the lower ATP concentration and the higher oxygen consumptions observed at day 2 in N-, P-, and S-deprived roots compared to the control may trigger PCD by perturbations in energy status of the root (Siyiannis et al., 2012).

Apart from roots, Maniou et al. (2014) reported that aerenchyma was formed in the lamina (LA) of the second leaf in maize under sulfate deprivation. In maize leaves, there is a cooperation between bundle sheath cells (BSC) and mesophyll cells (MC) for sulfate reduction and glutathione synthesis (Burgener et al., 1998; Kopriva and Koprivova, 2005; Kopriva, 2006). Plants utilize sulfate for synthesis of various organic compounds (such as cysteine, cystine, methionine, lipoic acid, co-enzyme A, thiamine pyrophosphate, glutathione, biotin, adenosine-5′-phosphosulfate, 3-phosphoadenosine, and proteins) through a complex metabolic network (Leustek and Saito, 1999; Leustek et al., 2000; Grossman and Takahashi, 2001), and sulfate deficiency causes retarded and chlorotic growth of plants (Maruyama-Nakashita et al., 2003). The concentration of glutathione is dependent upon S-nutrition (Blake-Kalff et al., 1998).

The scope of this work was twofold. We aimed at investigating (i) whether maize produces aerenchyma in other organs under sulfate deprivation and to map the developmental progress of this aerenchyma, if any, under prolonged S-deprivation conditions, and (ii) whether sulfur allocation was in any relationship with this phene. To this end, 7-day-old maize plants were transferred to sulfate deprived nutrient solution against complete nutrient solution and the various organs were investigated for aerenchyma formation at the 10th and the 19th day of the treatment. Total sulfur and sulfate concentrations of each organ were determined and organic sulfur was calculated by calculating the amounts of total sulfur and sulfate per organ and day and subtracting, whilst a number of morphometric parameters was measured (i.e., organ dry mass, organ length, specific root length, leaf surface area, specific surface area, as well as section areas of the mesocotyl’s stele, aerenchyma and cortex).

## MATERIALS AND METHODS

### PLANT MATERIAL AND HYDROPONICS SET UP

Maize (*Zea mays* “Cisko,” Syngenta Hellas) seeds were kept on wet filter paper, in the dark (28°C, relative humidity 76%) until germination. Four days later, the most uniform of those plants were selected and maintained in a hydroponic batch culture for 3 days in well-aerated distilled H_2_O. A controlled environment of 250 μmol photons m^-2^ s^-1^ photosynthetic photon flux density (PPFD) and a 14-h light photoperiod with day/night growth conditions at shoot base 28/23°C and RH 36/40% was used. Complete nutrient solution (control) contained 5 mM KNO_3_, 1 mM KH_2_PO_4_, 2 mM Mg(NO_3_)_2_, 2.5 mM CaSO_4_, 1 mM MgSO_4_, 0.07 mM EDTAFeNa, 4 mM Ca(NO_3_)_2_, 0.9 μM ZnCl_2_, 30 μM H_3_BO_3_, 0.9 μM CuCl_2_, 0.5 μM MoO_3_ and 20 μM MnCl_2_. S-deprived nutrient solution (–S) contained 5 mM KNO_3_, 1 mM KH_2_PO_4_, 2 mM Mg(NO_3_)_2_, 0.07 mM EDTAFeNa, 4 mM Ca(NO_3_)_2_, 0.86 mM CaCl_2_, 0.9 μM ZnCl_2_, 30 μM H_3_BO_3_, 0.9 μM CuCl_2_, 0.5 μM MoO_3_ and 20 μM MnCl_2_. At d7 and for the next 19 days, hydroponic batch cultures were run by using the respective nutrient solutions. All nutrient solutions were constantly aerated and replaced every 3 days.

### HISTOLOGICAL STUDY

Samples were fixed in formaldehyde/glutaraldehyde fixative (3.7%/0.25% v/v) and dehydrated through an ethanol dehydration series at room temperature. After dehydration, samples were transferred into paraffin through xylene as a paraffin/xylene infiltration. Tissues samples were embedded in paraffin blocks and paraffin sections of a thickness of 15 μm were taken, using a standard rotary microtome Leica Jung 2025. Paraffin sections were mounted to glass microscope slides coated with poly-L-lysine. Mounted section were deparaffinized in two changes of xylene and hydrated by transferring slides first to an ethanol:xylene mixture then to a graded series of decreasing ethanol concentrations. Sections were then stained using Johansen’s Safranin and Fast Green protocol (Ruzin, 1999). Sections were viewed and photographed using a Zeiss Axiolab HBO 50 light microscope, and analyzed by using the ImageJ software.

### TRANSPIRATION RATE DETERMINATION

For the calculation of transpiration rates, at d9 and d18 of the treatment four vessels of 1 L each were used, covered with aluminum foil. Nutrient solution was added to each vessel to a final weight of 1100 g, as follows: C nutrient solution in the first vessel and C nutrient solution plus 1 plant in the second one, –S nutrient solution in the third vessel and –S nutrient solution plus 1 plant in the fourth one. After 24 h the vessels were weighed, and the mass of the water lost was recorded. Three repetitions of each determination were accomplished.

### CHEMICAL ANALYSIS

Fresh weight per organ was recorded, the plant parts were oven-dried at 80°C, and the dry weight was recorded. Then, composite samples of the appropriate dry mass were ground to pass a 40 mesh screen using an analytical mill (IKA, model A10) prior to chemical analysis (Mills and Jones, 1996). Sulfate concentration was determined by extracting the ovendried samples with 2% (v/v) acetic acid aqueous solution and by analyzing with a turbidimetric method (Sörbo, 1987; Miller, 1998). Total sulfur concentration (S_tot_) was determined after dry-ashing at 600°C (Astolfi et al., 2003). The ash was dissolved in 2% (v/v) acetic acid aqueous solution, filtered through Whatman No. 42 paper, and total sulfate was determined turbidimetrically (Sörbo, 1987; Miller, 1998). S_tot_ and sulfate amounts per organ and day were calculated from their concentrations, and organic sulfur (S_org_) per organ and day was calculated by subtracting sulfate amount from S_tot_ amount.

### STATISTICAL ANALYSIS

Each treatment (C vs. –S) was repeated three times, by conducting three separate hydroponic experiments. Within each repetition, a number of plants was taken, which ensured an adequate amount of dry mass, and the composite sample was used for chemical analyses. In accordance with the above, three composite samples were separately analyzed. The comparisons between the corresponding –S and C values were submitted to *t*-test variance analysis with two-tailed distribution and two-sample equal variance, at *p* ≤ 5%. Where the differences between means of C and –S samples were statistically significant, the percentage of the relative change is marked with asterisk. Regression analysis was performed using the R platform (R Development Core Team, R Foundation for Statistical Computing, Vienna, Austria), according to Crawley (2007).

## RESULTS

### THE SECOND –S LAMINA FORMED AERENCHYMA MAINLY DISTRIBUTED ALONG THE MIDDLE REGION OF LEAF AXIS

**FIGURE 1:**
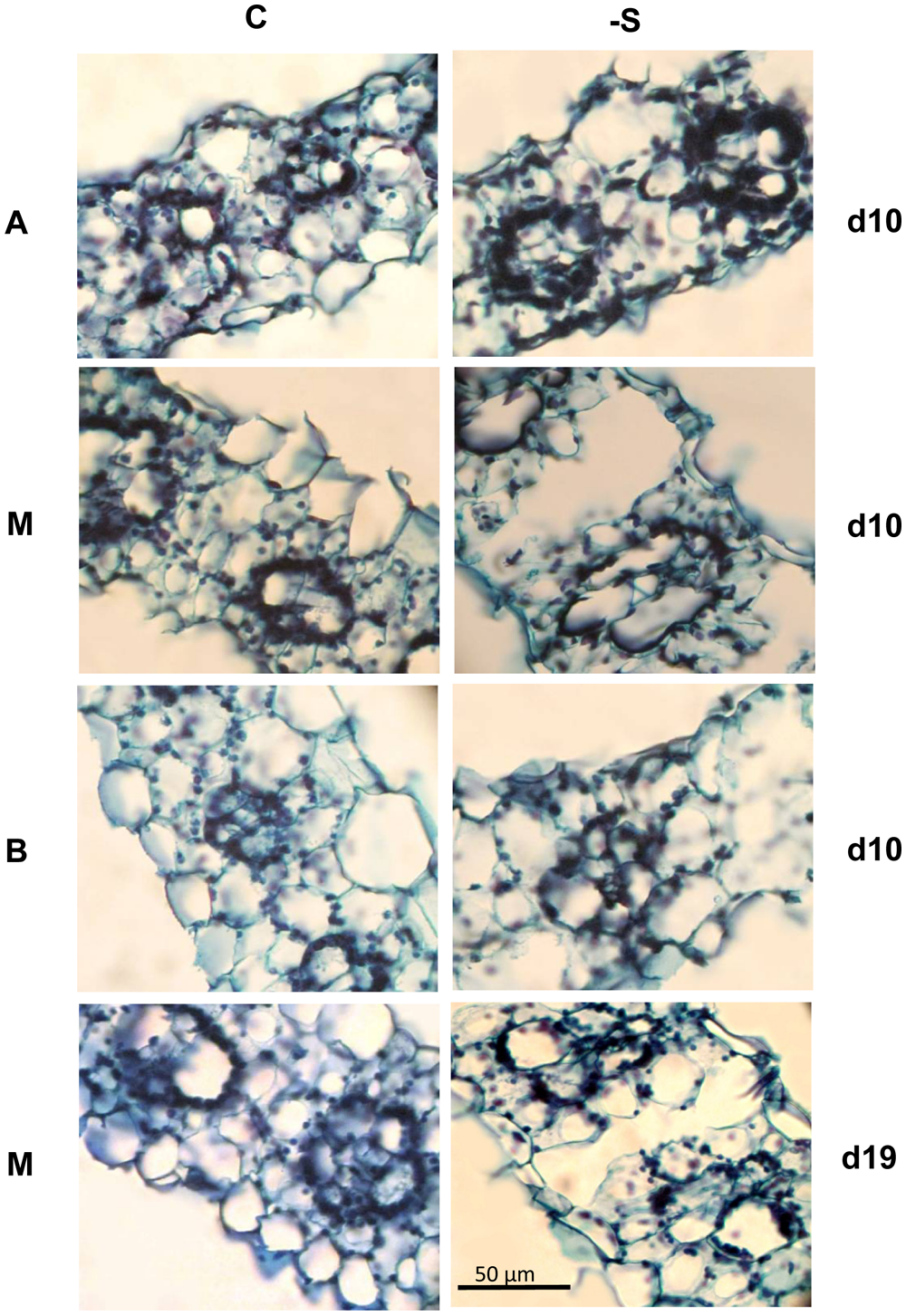
**Cross sections taken from the second lamina (LA) of plants under full nutrition (C ) or S-deprivation (-S), at d10 and d19 of the treatment.** A, the LA’s apex; M, middle; or B, base.

**FIGURE 2:**
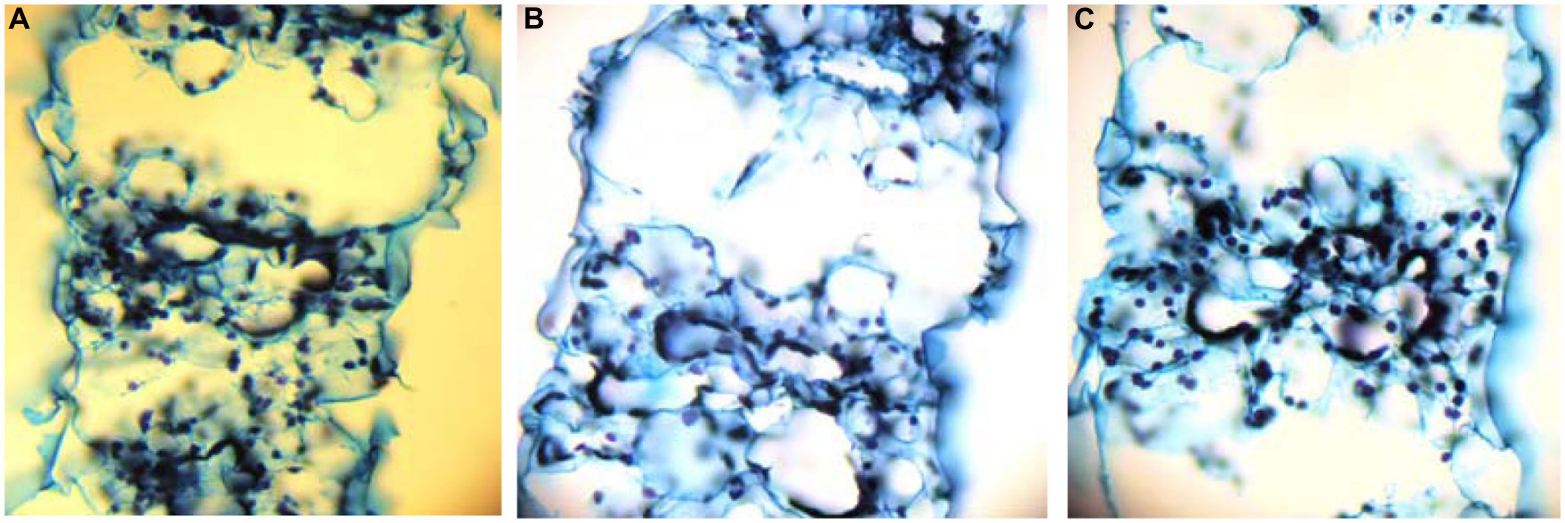
**Cross sections taken from the middle of the second LA’s axis, that depict the three variations of aerenchyma connecting epidermis to epidermis **(A)**, stoma to stoma **(B)**, or stoma to epidermis (C)**.

Aerenchyma was found to be formed in the LA of the second leaf, and its distribution within the LA was not uniform. At d10 under the deprivation, larger substomatal cavities appeared in the LA’s upper part compared to control. In the middle of the LA’s axis, large cavities appeared between the vascular bundles instead of MC (**Figure 1**). Aerenchyma was extended for the abaxial to the adaxial epidermis and three variations of this motif were distinguished: (i) from epidermis to the opposite epidermis, (ii) from epidermis to the opposite stomatal cavity, and (iii) from stomatal cavity to the opposite stomatal cavity (**Figure 2**). The third variation was less frequent, whilst the other two ones appeared with almost the same frequency. The first variation was found in the LA’s base, too, with reduced frequency. At d19 under the deprivation, aerenchyma was found only in the middle region of the leaf axis. The sheath (SH) of the second leaf did not form aerenchyma in any position.

### AT D10 MORE SURFACE AREA WAS PRODUCED IN –S LEAVES PER UNIT DRY MASS

During the 10th day of the treatment, –S plants presented per plant statistically the same transpiration rate (25 g H_2_O plant^-1^ d^-1^), although the invested dry mass of the aerial part was less than control by 20.7% and the total surface area of leaves (SHs + LAs) was more by 33.6%, without any change in length. In contrast, transpiration rate per plant at d19 was reduced by 68.2%; the invested dry mass of the aerial part, the total surface area and the total length of the leaves were all less than control by 61.8, 58.5, and 36.6%, respectively.

Under the deprivation, the variation of these morphometric parameters within leaves (i.e., SHs and LAs) with leaf position presented targeted changes. At d10, dry mass reduced in the upper SH and the two upper LAs, whilst that of the second leaf remained unaffected; surface area increased in two upper SHs and four upper LAs, starting from the second leaf’s SH and LA; length increased in the upper SH and the two upper LAs, whilst that of the second leaf remained unaffected (**Table 1**). Changes in dry mass and surface area by S-deprivation were more significant in SHs than in LAs. The LA’s width in the middle of the second leaf axis was reduced by 13.5% at the central bundle and increased by 16% at the bundles near the edge with no significant changes inbetween. SH of the second leaf presented significant increase of width only in the central bundle (**Table 2**).

**Table 1:** The effect of S-deprivation treatment (-S) on the partitioning of dry mass, surface area, and length between sheath (SH) and lamina (LA), according to leaf position and day of treatment.

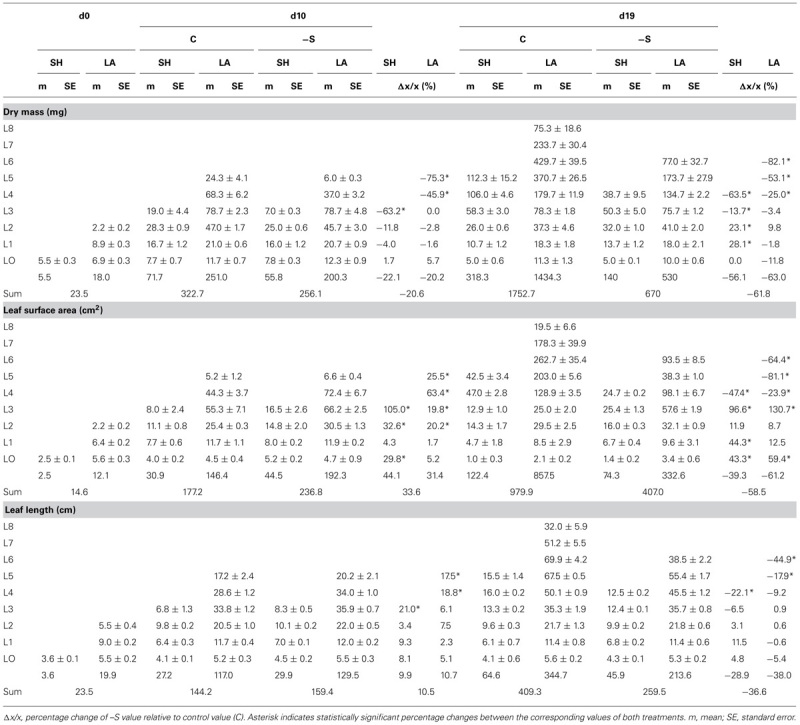

**Table 2:** The effect of S-deprivation treatment (-S) on SH and LA width allocation along the second leaf according to day of treatment.

		Width (μm)								
		C			-S			Δx/x (%)		
		CE	IN	ED	CE	IN	ED	CE	IN	ED
d0	LA	93	50	40						
d10	LA SH	766 536	159 354	94 147	663 622	159 345	112 156	-13.5 16.0	-0.2 -2.4	19.8 5.6
d19	LA SH	801 661	170 370	107 161	729 639	169 384	106 144	-9.0 -3.3	-0.5 3.8	-0.3 -10.5

*Δx/x, percentage change of –S value relative to control value (C). CE, width at the central bundle; IN, width at five (intermediate) bundles from the central one; ED, width at three bundles from the leaf edge.*

At d19, the dry mass of the first and second leaf’s SH increased, whilst that of the rest three decreased, along with all four upper LAs(**Table 1**); the surface area of both SHs and LAs increased up to the third leaf with the exception of the second LA which remained unaffected, whilst all SHs and LAs above the third leaf decreased; the length of SHs and LAs remained unaffected up to the third and fourth leaf, respectively, whilst all others decreased (**Table 1**). No significant changes were observed in the LA’s width breadthwise in the middle of the leaf axis (**Table 2**). –S LAs were increasingly affected with increasing position and more compared to –S SHs, in contrast to the observed effect at d10.

**FIGURE 3:**
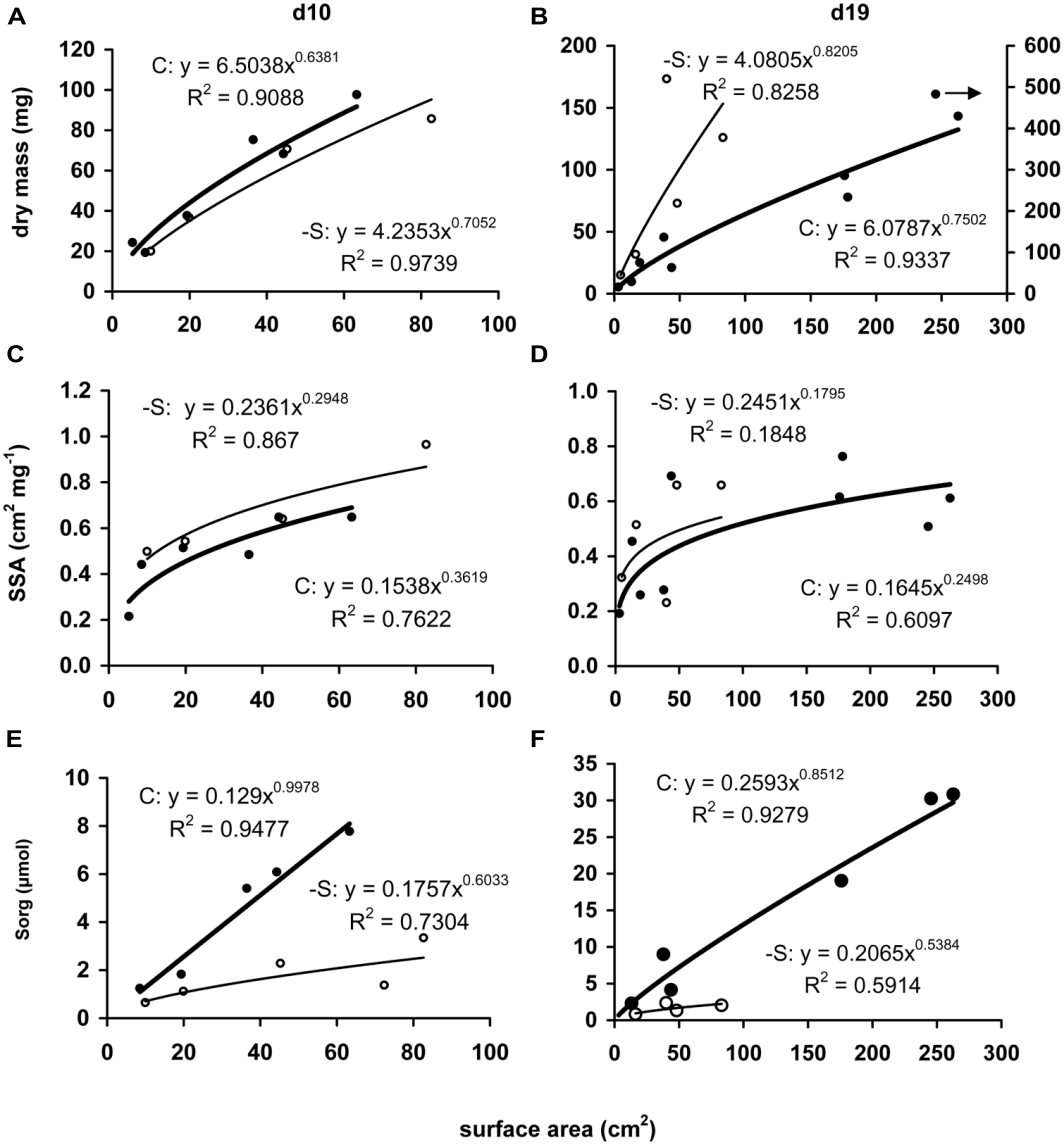
**Correlations of leaves’ dry mass **(A,B)**, specific surface area (SSA; **C,D**), or accumulated amount of organic sulfur (Sorg; **E,F**) with the corresponding surface area.** Each point respresents a leaf (sheath + lamina) and each value has been expressed per leaf. Full circle and bold line, control treatment; open circle and thin line: S-deprivation. Arrow indicates the corresponding y-axis.

Leaf surface area was positively correlated with increasing leaf dry mass and the correlation has been followed by applying a power function. In –S leaves at d10, more surface area had been produced with the same amount of dry mass (**Figure 3A**) and the exponent (n) of the power function was higher by 10.5% which reflects lower deviation of the trend line from linearity. Correlating surface area with specific surface area (i.e., surface area per dry mass, SSA), the SSA of –S leaves was higher and the surface more expanded compared to that of the control leaves (**Figure 3C**), the exponent being lower by 18.5%. At d19, although both trend lines presented higher exponents than the corresponding ones at d10, the exponent n_-S_ shared the same relative change with d10, as it was higher than n_C_ by 9.4%. In contrast to d10, at d19 more dry mass was invested in the –S leaves with no significant increase in surface area (after d10, –S leaves did not expand their surface area over 80 cm^2^, **Figure 3B**). The correlation between SSA and surface area did not produce differences compared with the control leaves and the power function provided a poor fit (**Figure 3D**), whilst control leaves fluctuated around 0.6 cm^2^ mg^-1^ dry mass. As regards the invested organic sulfur, in control leaves at d10 it was in almost linear relationship with their surface area (*n* = 0.9978), whilst the deprivation caused a deviation from the linearity by 39.5%, which suggests that the invested organic sulfur was proportionally less with the produced surface area (**Figure 3E**). At d19, the same picture emerged (**Figure 3F**); the –S leaves presented a hectic progress with poorer relationship between organic sulfur and surface area, and reduction of the exponent by 36.7%.

### –S MESOCOTYL FORMED AERENCHYMA NEAR THE SCUTELAR NODE

**FIGURE 4:**
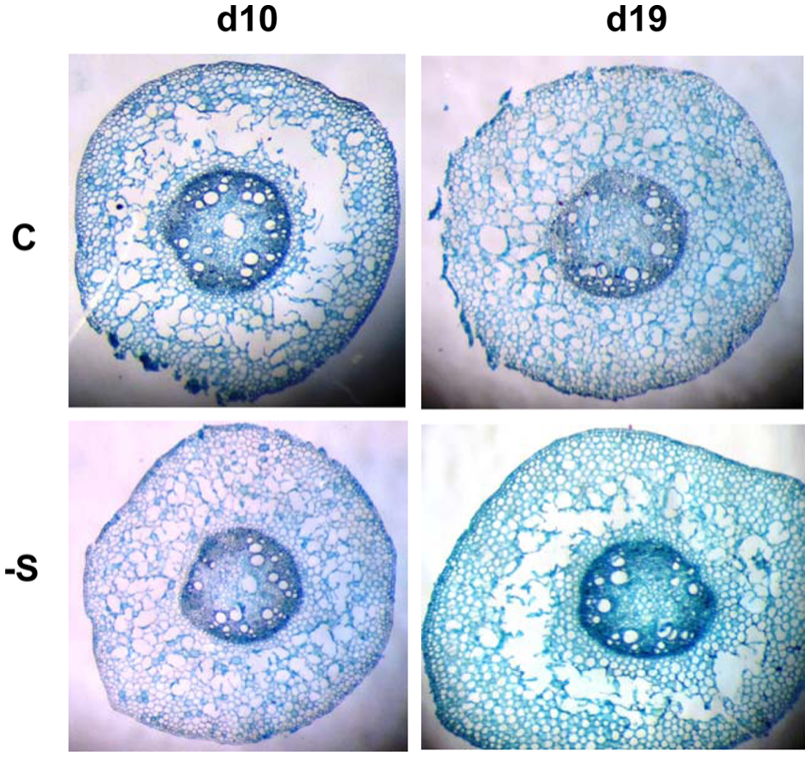
**Aerenchyma formation in mesocotyl near the scutelar node, at d10 and d19 of the sulfate deprivation (-S) and control (C) treatment**.

Near the crown, the mesocotyl (Mc) did not present aerenchyma, whilst near the seed (Ms) aerenchyma was present even under full nutrition (**Figure 4**). At d10, 23.7% of the Ms section’s area was occupied by aerenchyma in the cortex and the aerenchymatous area remained unchanged thereafter resulting in less percentage contribution (17.2%). In control plants, Mc section area was larger than Ms. At d10, the deprivation resulted in the formation of less aerenchyma than control, in favor to the formation of cortex. At d19 under the deprivation, Ms was of the same size as Mc and the aerenchymatous area was 26.5% larger than control, whilst the percentage contribution of the aerenchymatious area to the whole one was that of d10 (**Table 3**). Mesocotyl roots (MR) were not uniformly distributed along mesocotyl axis. Most of them were located at Ms, i.e., near the scutelar node, a location that coinsides with the presence of aerenchyma development in the cortex. Mesocotyl length remained statistically unchanged in both treatments.

**Table 3:** The cross section area of mesocotyl near the crown (Mc) or near the seed (Ms) and the area of aerenchyma, cortex, and stele at day 10 and 19 of the treatment.

	C				-S			
	Section area	Stele	Aerenchyma	Cortex	Section area	Stele	Aerenchyma	Cortex
**d10**
Mc	mm^2^	2.46	0.45	0	2.01	2.63	0.4	0	2.23
	%	100.0	18.3	0.0	81.7	100.0	15.2	0.0	84.8
Ms	mm^2^	2.07	0.33	0.49	1.25	2.08	0.3	0.34	1.44
	%	100.0	15.9	23.7	60.4	100.0	14.4	16.3	69.2
**d19**
Mc	mm^2^	3.76	0.74	0	3.02	3.6	0.64	0	2.96
	%	100.0	19.7	0.0	80.3	100.0	17.8	0.0	82.2
Ms	mm^2^	2.85	0.43	0.49	1.93	3.59	0.47	0.62	2.5
	%	100.0	15.1	17.2	67.7	100.0	13.1	17.3	69.6

*C, control treatment; -S, S-deprivation treatment.*

### THE ROOT SYSTEM RESPONDED DIFFERENTIALLY TO S-DEPRIVATION

**FIGURE 5:**
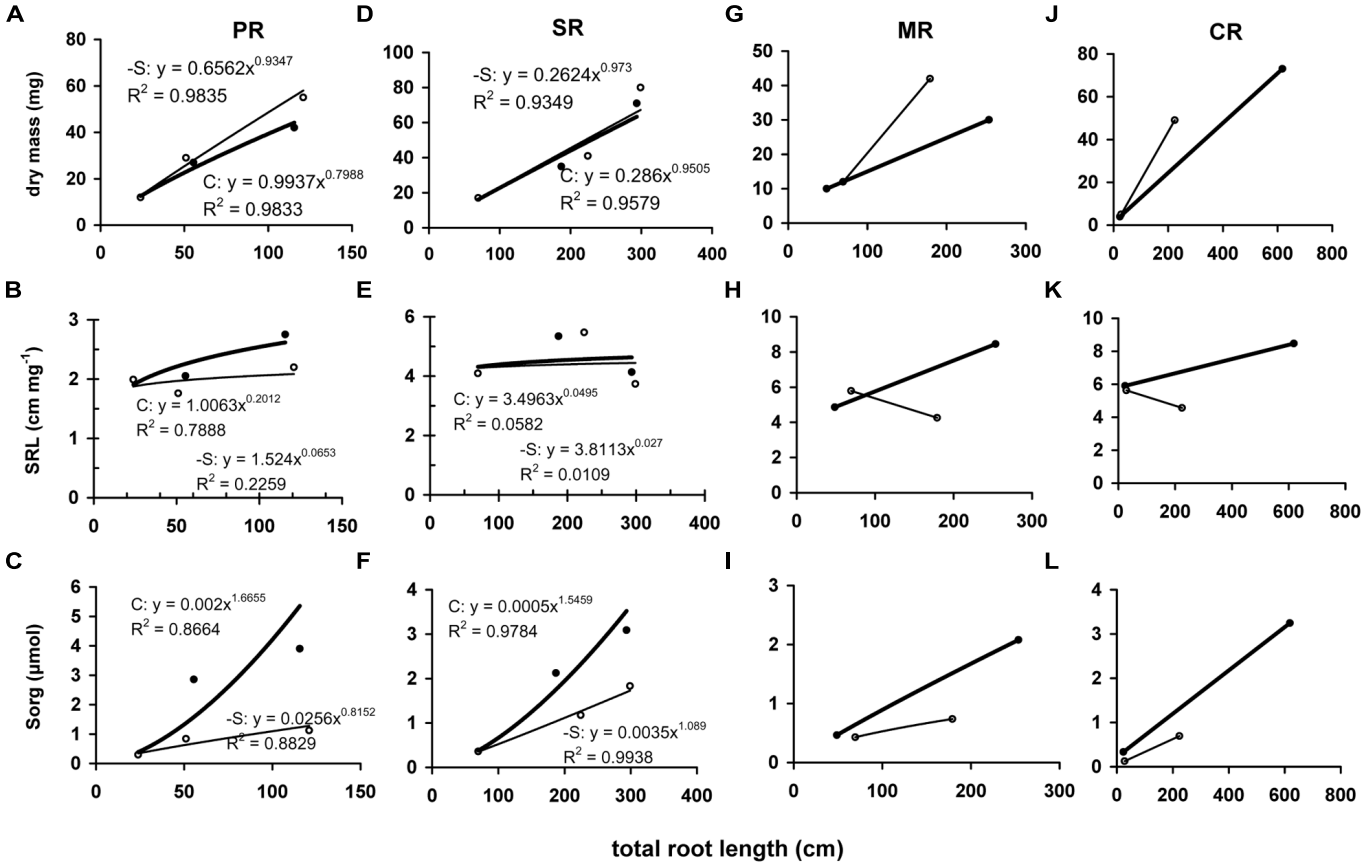
**Correlation of roots’ dry mass **(A,D,G,J)**, specific root length (SRL; **B,E,H,K**) or accumulated amount of organic sulfur (Sorg; **C,F,I,L**) with the corresponding total length of each root type.** Each point represents a root type (PR, primary root; SR, seminal roots; MR, mesocotyl roots; CR, crown roots) and each value has been expressed per organ. Full circle and bold line, control treatment; open circle and thin line: S-deprivation.

At d10, the dry mass that was allocated between root system and the aerial part was increased in the root system by 19.9% and decreased in the aerial part by 20.5%. In contrast, at d19 a reduction by 58.8% was observed in the aerial part and an increase by 11.8% in the root system. The root system was composed by four root types: a primary root (PR), seminal roots (SR), MR, and up to three whorls of CR. A typical composition of control plants included five root axes (1 PR and 4 SR) at d0, 10 root axes (1 PR, 4 SR, 2 MR, 3 CR1) at d10 and 20 root axes (1 PR, 4 SR, 4 MR, 4 CR1, 3 CR2, 4 CR3) at d19. The derpivation altered the number of axes within root type and reduced the total number of axes. At d10, 12 axes were present (1 PR, 5 SR, 3 MR, 3 CR1), whilst at d19 14 axis were present (1 PR, 5 SR, 3 MR, 3 CR1, CR2). Total root length was linearly correlated with dry mass and during the deprivation all root types but SR increased the invested dry mass differentially (**Figures 5A,D,G,J**). At d10, -S PR, SR, MR, and CR presented specific total root length (SRL) of 2 (no change), 5 (no change), 6 (increase by 40%), and 6 (no change) cm mg^-1^, respectively, whilst at d19, the corresponding values were 2 (decrease by 20%), 4 (no change), 4 (decrease by 50%), and 4 (decrease by 50%) cm mg^-1^, respectively (**Figures 5B,E,G,K**). The amount of organic sulfur that invested in each root type increased linearly with total root length, with differential slope. This held true for the –S roots, with significantly reduced slope (**Figures 5C,F,I,L**).

### REDUCED SULFUR ALLOCATION AMONG ORGANS FOLLOWED THAT OF DRY MASS IN A PROPORTIONAL FASHION

Although the S-deprivation treatment excluded sulfate anions, accumulations of total sulfur and sulfate were determined (**Table 4**). To explain this, the maximum possible influx of sulfate was calculated as impurities given by the production company for each reagent used for the preparation of the nutrient solution, although the reagents were of analytical grade. The calculated influx was in accordance with the difference between the determined amounts and that provided by the seed.

**Table 4:** Total sulfur and sulfate concentration in the organs of maize plants at days 0, 10, and 19 of the treatment.

	d0	d10	d19
		C	-S		C	-S	
	Mean SD	Mean SD	Mean SD	Δx/x	Mean SD	Mean SD	Δx/x
	μmol gDM^-1^		μmol gDM^-1^	%		μmol gDM^-1^	%
**Total sulfur concentration per organ and day**
L6					(3) 71.8 ± 2.8	17.3 ± 2.9	-76.0*
L5					62.6 ± 0.5	15.4 ± 1.5	-75.5*
L4		(2) 89.2 ± 2.2	37.1 ± 0.5	-58.4*	66.6 ± 3.1	13.6 ± 1.0	-79.6*
L3		79.4 ± 5.1	39.0 ± 0.1	-50.9*	65.7 ± 4.6	16.1 ± 1.2	-75.6*
L2		71.9 ± 1.6	32.5 ± 0.3	-54.8*	65.5 ± 3.6	18.1 ± 1.6	-72.3*
L1	(1) 50.5 ± 1.1	48.3 ± 3.3	30.7 ± 0.6	-36.4*	79.4 ± 6.9	25.3 ± 2.9	-68.1*
LO	42.4 ± 1.4	65.7 ± 1.3	32.4 ± 0.6	-50.7*	49.8 ± 2.7	30.8 ± 3.1	-38.1*
Col	45.1 ± 0.2	80.3 ± 4.1	33.8 ± 2.9	-57.9*			
C + M	37.3 ± 0.1	95.1 ± 1.6	29.7 ± 0.5	-68.8*	96.8 ± 8.0	19.0 ± 0.8	-80.3*
CR		154.3 ± 6.0	32.8 ± 1.0	-78.7*	150.2 ± 2.8	18.3 ± 2.0	-87.8*
MR		190.2 ± 6.0	39.6 ± 1.1	-79.2*	189.9 ± 3.7	20.8 ± 1.3	-89.0*
Seed	22.5 ± 0.5	47.0 ± 0.9	28.7 ± 0.6	-38.9*	66.7 ± 2.6	33.8 ± 1.3	-49.3*
SR	29.6 ± 0.8	209.6 ± 26.1	43.7 ± 0.9	-79.2*	219.3 ± 27.9	25.3 ± 2.1	-88.5*
PR	29.7 ± 0.4	215.5 ± 22.3	43.1 ± 0.6	-80.0*	180.7 ± 1.1	25.7 ± 1.7	-85.8*
**Sulfate concentration per organ and day**
L6					(3) 4.6 ± 1.1	5.7 ± 2.3	23.9
L5					21.5 ± 2.4	7.3 ± 1.3	-66.0*
L4		(2) 41.5 ± 3.1	6.7 ± 1.4	-83.9*	20.9 ± 3.3	6.8 ± 3.0	-67.5*
L3		32.2 ± 0.4	4.6 ± 0.8	-85.7*	14.6 ± 3.4	4.9 ± 1.7	-66.4*
L2		19.3 ± 1.2	4.4 ± 0.3	-77.2*	3.7 ± 0.5	3.1 ± 0.8	-16.2
L1	(1) 26.8 ± 9.3	6.4 ± 3.5	4.8 ± 1.0	-25.0	17 ± 4.4	3.5 ± 0.8	-79.4*
LO	3.5 ± 1.6	43.5 ± 2.5	7.1 ± 3.3	-83.7*	3.5 ± 0.7	3.4 ± 1.8	-2.9
Col	9.3 ± 3.0	5.5 ± 0.3	7.7 ± 4.3	40.0			
C + M	6.3 ± 1.1	50.3 ± 2.3	4.8 ± 0.2	-90.5*	58.9 ± 16.2	3.1 ± 1.1	-94.7*
CR		70.6 ± 18.1	7.8 ± 4.1	-89.0*	105.7 ± 17.5	4.1 ± 1.2	-96.1*
MR		143.6 ± 25.0	3.8 ± 0.2	-97.4*	120.6 ± 14.4	3.2 ± 1.0	-97.3*
Seed	6.3 ± 1.2	30.2 ± 3.5	5.6 ± 3.0	-81.5*	31 ± 2.9	3.6 ± 0.2	-88.4*
SR	8.5 ± 0.3	148.9 ± 25.3	15.0 ± 4.5	-89.9*	175.8 ± 39.8	2.4 ± 0.7	-98.6*
PR	5.1 ± 1.9	109.7 ± 24.9	14.3 ± 1.4	-87.0*	132.1 ± 15.3	5.3 ± 0.6	-96.0*

*The deprivation started at d7 from sowing. Δx/x, the relative percentage change in the corresponding concentration due to the deprivation; SD, standard deviation; L, leaf; Col, coleoptile; C + M, crown plus mesocotyl; PR, primary root; SR, seminal roots; MR, mesocotyl roots; CR, crown roots. Asterisk indicates statistically significant differences.*

*(1) includes L1, L2, and SAM*

*(2) includes L4, L5, and SAM*

*(3) includes L6, L7, L8, and SAM*

**FIGURE 6:**
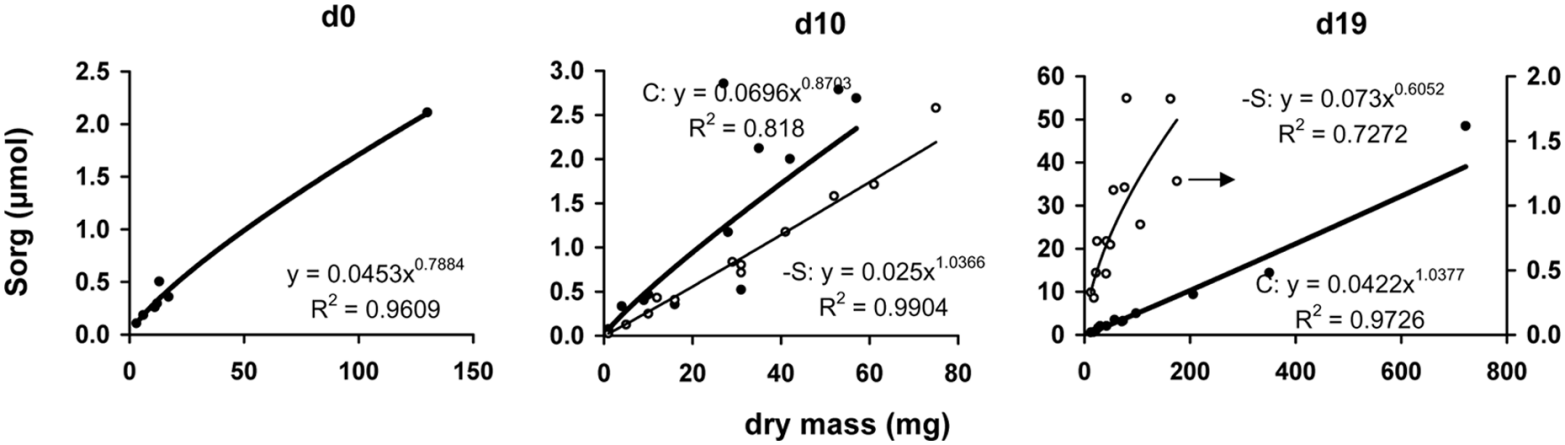
**The relationship between the dry mass of each organ and the corresponding amount of organic sulfur at days 0, 10, and 19 of the treatment.** Full circle and bold line, control treatment, open circle and thin line, S-deprivation treatment; At d19, the regression line of -S treatment represents only the points that followed the power function relationship (see text for Discussion). The arrow indicates the corresponding axis.

The amount of organic sulfur allocated in each organ presented very high positive correlation with allocated dry mass in this organ (**Figure 6**). Considering linear relationship, the calculated mean slopes were 24.0, 49.0, and 50.5 μmol gDM^-1^ under full nutrition at days 0, 10, and 19, vs. 28.2 and 18.9 μmol gDM^-1^ at days 10 and 19 under S-deprivation, respectively.

## DISCUSSION

It is known that crown (or nodal) roots create aerenchyma under certain conditions, sulfate deprivation among them. This is a trophic aerenchyma and the basic difference compared to hypoxic aerenchyma is that the trophic one does not form a continuum from shoot to root (Bouranis et al., 2006). This work provides new insights into the formation of trophic aerenchyma in maize. The stimulus for this work was the fact that at d10 under the deprivation the second leaf was characterized by both more surface area (by 24.1%) and less dry mass (by 6.7%) than control. The second leaf along with the first CR were the organs that were just emerging at the onset of the deprivation. Therefore, the working hypothesis was that cells are eliminated with PCD in order to invest the produced dry mass towards increasing leaf surface area along with root length for more efficient acquisition of resources under the nutritional imbalance.

The aerenchymatous CR are attached to crown and crown found to contain no aerenchyma. This held true also for the SH of the second leaf; it was not aerenchymatous, too. Instead, the LA of the second leaf presented aerenchyma formation. The allocation of lacunae within the LA presented a pattern: the enlarged substomatal spaces found at the upper part of the LA progressively became large spaces by eliminating the whole parenchymatous tissue from abaxial epidermis to the opposite adaxial epidermis between bundles. This pattern was profound in the middle part of the LA, progressively faded towards its base. Nine days later, the percentage of aerenchyma was less compared to that at d10 under the deprivation. This fact suggests that aerenchyma formation did not enlarge with the LA’s growth. Therefore, the accumulation of dry mass during the following days resulted in the reduction of the area that is occupied by lacunae, thus reducing its percentage contribution to total area of the cross section. Breadthwise the size of bundles reduces; as a result bundles of maize are distinguished to large, intermediate and small with different function (Fritz et al., 1989). We also know that the central bundle of the second LA under sulfate deprivation is more robust than that of control and lignification has been stimulated, obviously to mechanically support the aerenchymatous tissues (Bouranis et al., 2007a). In a previous work where the impact of sulfate deprivation on stomatal conductance, transpiration rate, and photosynthetic rate were examined, the LA’s surface area was not found to be increased at d10 (Bouranis et al., 2012). The plants were receiving 170 μmol photons m^-2^⋅s^-1^ PPFD, whilst in this work the photon flux was adjusted to 250 μmol photons m^-2^⋅s^-1^. Astolfi et al. (2001) have reported that an increase in irradiance accelerated the development of the S deficiency. Obviously, the combination of increased irradiance by 47% with the sulfate deprivation forced the leaf to form localized aerenchyma. It is noteworthy that dry mass partitioning between SHs and LAs was of the order 1:4 (SH:LA) and this ratio does not seem to alter by the deprivation. This held true for surface area and length partitioning between SHs and LAs. Instead, an internal arrangement took place at the expence of the younger leaves (both SHs and LAs) above the second leaf. The effect of sulfate deprivation on distribution profile of stomatal conductance and its interrelations to transpiration rate and water dynamics in young maize LAs have been examined (Bouranis et al., in press). Under the experimental conditions of this work, both control or, –S plants presented the same transpiration rate at the whole plant level. The finding that aerenchyma was in fact the extension of the stomatal cavities or in direct connection with them, suggests one more role of aerenchyma formation in leaves; to accelerate nutrient absorption and transport towards the aerial part.

At the same time and apart from aerenchyma in the cortex of –S CR, aerenchyma was also found in –S mesocotyl near the scutelar node. Interestingly, this was also the case for the mesocotyl of control plants and the deprivation reduced aerenchyma formation by 7.4% (**Table 3**). In both cases, the location of aerenchyma along the mesocotyl axis coincided with the presence of MR, which were not uniformly allocated along mesocotyl axis. Our data suggest that mesocotyl cortical aerenchyma supports the MR, which are lateral relative to the mesocotyl axis; mesocotyl behaves as a root axis. Under the deprivation, the root system contained five SR (instead of four under full nutrition) and three MR (instead two at d10 and four at d19 under full nutrition), a finding that probably explains the reduced aerenchyma formation in the mesocotyl (less root axes with more invested dry mass). All root axes that are attached to scutelar node presented no aerenchyma at the vicinity of the scutelar node, i.e., their base, which is sensible because the scutelar node is trafficing center. This is another proof that this aerenchyma is not formed for the transfer of oxygen, i.e., is not a ventilating trait. These findings suggest that trophic aerenchyma formation in maize follows a strategy at the whole plant level. This strategy includes the protection of vital tissues by preventing PCD in the crown and in the scutelar node. The latter is temporary during plant’s development, because it may be destroyed soon, whilst crown is a vital organ and as such is protected. Thus the basal zones of the attached organs are not subject to aerenchyma formation. It is considered that PCD is involved in nutrient cycling; it has been shown that this mechanism plays a role in nitrogen remobilization and because it is a non-specific mechanism it could also control remobilization of nutrients (Pottier et al., 2014).

Allocation has been conceptualized as a ratio-driven process. At any point in time a plant allocates the amount of available resources to different structures and allocation has been analyzed by means of power function. Considering allocation as a size-dependent process, the quantitative relationship between growth and allocation is called allometry. Size is represented by organ dry mass, which is affected by the deprivation. Plasticity in allocation is the alteration of the plant’s allometric developmental plan in response to the environment. Such an allometric approach of the sulfate deprivation’s impact on nutrient allocation has been applied in young maize plants (Bouranis et al., 2014) and this allometric approach has been used for the analysis of the data presented in **Figures 3, 5, and 6**. This analysis strongly suggested that in plants under full nutrition the allocation of organic sulfur among organs followed that of dry mass in a proportional fashion and this held true for –S organs at d10 under the deprivation. At d19, the –S leaves L4, L5, and L6 diverted from linearity (these leaves were deployed during the deprivation). In this work, the deprivation started immediately after the transfer of seedlings from water to nutrient solution. Thus, the existing reduced sulfur came from the seed reserves plus the impurities of the used salts. It is quite impressive that although there is available sulfate, this amount was not used (**Table 4**), which suggests that the needs of the reduced sulfur were balanced under the circumstances and this is documented by the correlations between organic sulfur concentrations and specific surface area or specific root length.

## Conflict of Interest Statement

The authors declare that the research was conducted in the absence of any commercial or financial relationships that could be construed as a potential conflict of interest.
